# Forgotten Variables in Gerontological Evaluation of Religiosity and Spirituality

**DOI:** 10.1093/geroni/igaa057.1990

**Published:** 2020-12-16

**Authors:** Alex Bishop, Rosemary Blieszner

## Abstract

Despite the fact that religiosity and spirituality are commonly cited as essential elements of physical and mental health functioning in old and very old age, the conceptualization of religiosity and spirituality continues to present a quandary in contemporary gerontological inquiry and assessment. Identification of variables that best capture and truly define religious and spiritual constructs has remained relatively inconclusive over the past few decades. Recently, there has been a renewed interest among gerontologists to identify underlying religious and spiritual variables using alternative qualitative and quantitative methods of evaluation. Such methods have been used to further disentangle and understand the uniqueness of religion and spirituality as variables for studying human aging. This symposium will be used to communicate empirical results, methodological procedures, and conceptual insights concerning the identification of understudied and under-represented variables often absent within modern gerontological inquiry of religion and spirituality. Four presentations representing qualitative and quantitative studies will be used to report key religious and spiritual variables stemming from the context of personal narrative, oral storytelling, self-improvement behavior, and the disposition to seek forgiveness. Recommendations pertaining to future gerontological inquiry in the science of religion and spirituality, as well as applications within geriatric and gerontological practice will be highlighted and discussed.

